# Functional divergence and intron variability during evolution of angiosperm *TERMINAL FLOWER1* (*TFL1*) genes

**DOI:** 10.1038/s41598-017-13645-0

**Published:** 2017-11-01

**Authors:** Jian Gao, Bing-Hong Huang, Yu-Ting Wan, JenYu Chang, Jun-Qing Li, Pei-Chun Liao

**Affiliations:** 1 College of Forestry, Beijing Forestry University, No.35, Tsinghua East Rd., Haidian Dist., Beijing, 100083 People’s Republic of China; 2 Department of Life Science, National Taiwan Normal University, No.88, Sec. 4, Tingjhou Rd., Wunshan Dist., Taipei, 116 Taiwan, Republic of China; 3 Department of Horticulture, Chiayi Agricultural Experiment Branch, Taiwan Agricultural Research Institute No. 1, Nung-Kai-Chang, Lutsao township, Chiayi, 611 Taiwan, Republic of China

## Abstract

The protein encoded by the *TERMINAL FLOWER1* (*TFL1*) gene maintains indeterminacy in inflorescence meristem to repress flowering, and has undergone multiple duplications. However, basal angiosperms have one copy of a *TFL1*-like gene, which clusters with eudicot *TFL1/CEN* paralogs. Functional conservation has been reported in the paralogs *CENTRORADIALIS* (*CEN*) in eudicots, and *ROOTS CURL IN NPA* (*RCNs*) genes in monocots. In this study, long-term functional conservation and selective constraints were found between angiosperms, while the relaxation of selective constraints led to subfunctionalisation between paralogs. Long intron lengths of magnoliid *TFL1*-like gene contain more conserved motifs that potentially regulate *TFL1/CEN/RCNs* expression. These might be relevant to the functional flexibility of the non-duplicate *TFL1*-like gene in the basal angiosperms in comparison with the short, lower frequency intron lengths in eudicot and monocot *TFL1/CEN/RCNs* paralogs. The functionally conserved duplicates of eudicots and monocots evolved according to the duplication-degeneration-complementation model, avoiding redundancy by relaxation of selective constraints on exon 1 and exon 4. These data suggest that strong purifying selection has maintained the relevant functions of *TFL1/CEN/RCNs* paralogs on flowering regulation throughout the evolution of angiosperms, and the shorter introns with radical amino acid changes are important for the retention of paralogous duplicates.

## Introduction

**Figure 1:**
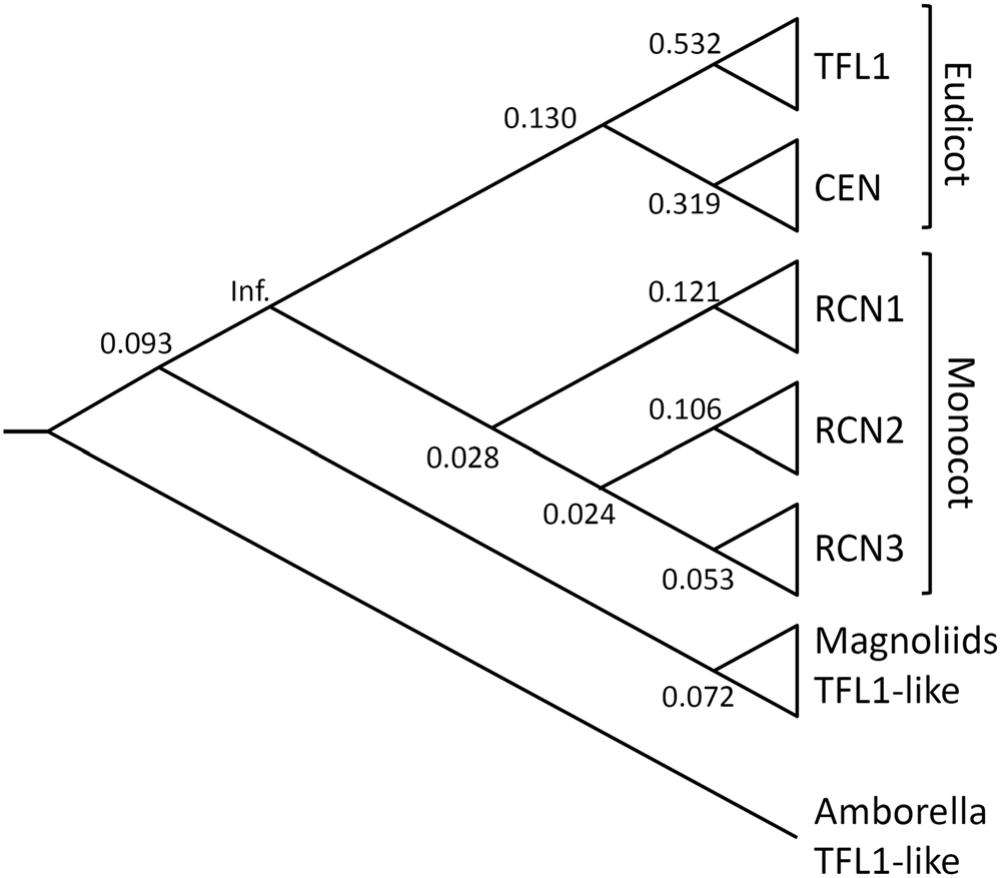
The hypothetical phylogenetic relationships of angiosperm *TFL1/CEN/RCN* paralogs. Values on the nodes indicate the *ω* of specific branches estimated under the free-ratio model, which suggest a pervasive purifying selection or selective constraints on the evolution of angiosperm *TFL1/CEN/RCNs* paralogs.

TERMINAL FLOWER1 (TFL1) is a member of the phosphatidylethanolamine-binding protein (PEBP) family. It represses flowering by counteracting the action of another PEBP protein, FLOWERING LOCUS T (FT), which promotes flowering^1^. The function of indeterminacy on shoot meristem of *Antirrhinum majus* suggests that the *CENTRORADIALIS* (*CEN*) gene is conserved and that its product is functionally identical to that of *TFL1* ^2,3^. *TFL1* and *CEN* are paralogous genes with conserved functions that involve the formation of inflorescences^4,5^ and the maintenance of indeterminacy in inflorescent meristems^6^. Most eudicot species possess low or one copy of *TFL/CEN* in their genomes^7^. The monocot *TFL1/CEN*-like paralogous genes, named *ROOTS CURL IN NPA* (*RCN1* and *RCN2)*, also share the same function and are expressed in a similar pattern in rice, whereas another duplicated gene, *RCN*3, may be a non-functional chimera^8^. The *TFL1*-like gene was also found in the transition in inflorescent indeterminacy/determinacy in *Phaseolus vulgaris* ^9^. The natural variation of *TFL1*-like gene may also be related to evolutionary transition of inflorescence architecture^10,11^. It has been suggested that gymnosperms lack orthologues of *FT* and *TFL1/CEN* ^7^. From functional analysis of the homologous *FT/TFL1*-like gene in gymnosperm, the repressive function of TFL1/CEN/RCNs in flowering is known to be plesiomorphic^12^. The angiosperm *TFL1/CEN/RCNs* paralogs are the result of multiple gene duplications: (1) after the divergence between basal angiosperms (*TFL1*-like) and eudicots + monocots (*TFL1/CEN/RCNs* paralogs), (2) two-time duplications resulting in *RCN1–*3 in monocots, and (3) gene duplication causing the divergence of *TFL1* and *CEN* in eudicots (phylogeny of angiosperms refers to *Amborella* Genome Project^13^, Fig. 1). The conserved function of TFL1/CEN/RCNs prevents redundancy or silencing by functional divergence^14,15^, which occurs by positive selection or through the relaxation of environmental constraints^15^.

Different expression patterns of duplicated *TFL1/CEN/RCNs* genes in *Arabidopsis* ^16^, apple^17^, tomato^18,19^, and tobacco^20^ tissues have been reported. Such differential expression was suggested as complementary functions (subfunctionalisation)^21,22^. Following functional divergence, genes normally experience a phase free from selective constraints^23^. Because of the conserved properties of *TFL1/CEN/RCNs* paralogs, poor resolution of nucleotide phylogeny^24^ cannot explain their divergence. Nevertheless, a single reciprocally switched amino acid could cause functional interconversion between *FT* (flowering activator) and *TFL* (flowering repressor)^25,26^. Therefore, a few changes in the amino acid sequence can alter protein function to escape the redundancy of duplicates^27^. Therefore, determining radical amino acid changes between *TFL1/CEN/RCNs* paralogs (the type-II functional divergence of Gu^28,29^) could be useful for predicting their functional divergence after duplication.

The functional conservation and divergence of paralogous genes is not only reflected in coding sequences, but also in exon-intron structure. Structural divergence is prevalent in duplicated genes and leads to functionally divergent paralogs^30^. Variable intron lengths could be relevant to functional compensation in coexisting paralogs^30^ and provide heterogeneous regulatory functions in duplicate^31–33^. Highly expressed genes have longer introns than genes expressed at low levels^33^. Exon length was also suggested to be associated with molecular functions in flowering development cf.^34^. The *Amborella trichopoda* genome (http://www.amborella.org/)^13^ enables the comparison of gene structure and sequence variation in *TFL1*-like gene between basal angiosperms, and monocot and eudicot angiosperms. Comparisons of gene structure and intron lengths may enhance our understanding of evolution and its relevance among paralogs.

Genetic diversity among *TFL1/CEN* homologs played a key role in the diversification of flowering plants^7,23^, which was probably driven by heterogeneous selective pressures on different gene regions. For example, strong selective sweeps in coding regions, and balancing selection of promoters were detected in *Arabidopsis* ^35^. Furthermore, epistatic selection was identified through a QTL closely linked to the *Arabidopsis TFL1* ^36^. In addition, latitudinal gradients adaptation was also inferred by nonsynonymous polymorphisms of *TFL1* ^37^. However, there have been limited studies focussed on the effects of selective pressures on *TFL1*/*CEN*/*RCNs* paralog duplication, as well as the *TFL1*-like gene in basal angiosperms. These functionally conserved paralogous gene duplicates may be subject to strong purifying selection pressures that constrain redundant functions, such as the floral-regulatory paralogs *SEPALLATA 1 *(*SEP1*) and *SEPALLATA 2* (*SEP2*), and *SHATTERPROOF 1* (*SHP1*) and *SHATTERPROOF 2* (*SHP2*)^38^. Selective constraints may be important in functionally redundant paralogous genes for buffering an organism’s phenotype against deleterious mutations^39^.

In this paper, a broad range of representative organisms from basal angiosperms, eudicots, and monocots were sampled to determine whether flowering plants exhibit divergent functions of *TFL1/CEN/RCNs* duplicates and how the selective pressure drove their evolution. General patterns of structural divergence in duplicated genes were analysed to represent the divergence/conservation of these paralogous genes. The aims of this research were to investigate (1) the evolution of intron variability in angiosperm *TFL1/CEN/RCNs* genes; (2) the effect of positive selection on angiosperm *TFL1/CEN/RCNs* coding sequences; and (3) the functional divergence between paralogs of angiosperm *TFL1/CEN/RCNs*, and thus infer the ancestral/derived type of *TFL1/CEN/RCN*s paralogs.

## Results

### Sequence length variation

**Figure 2:**
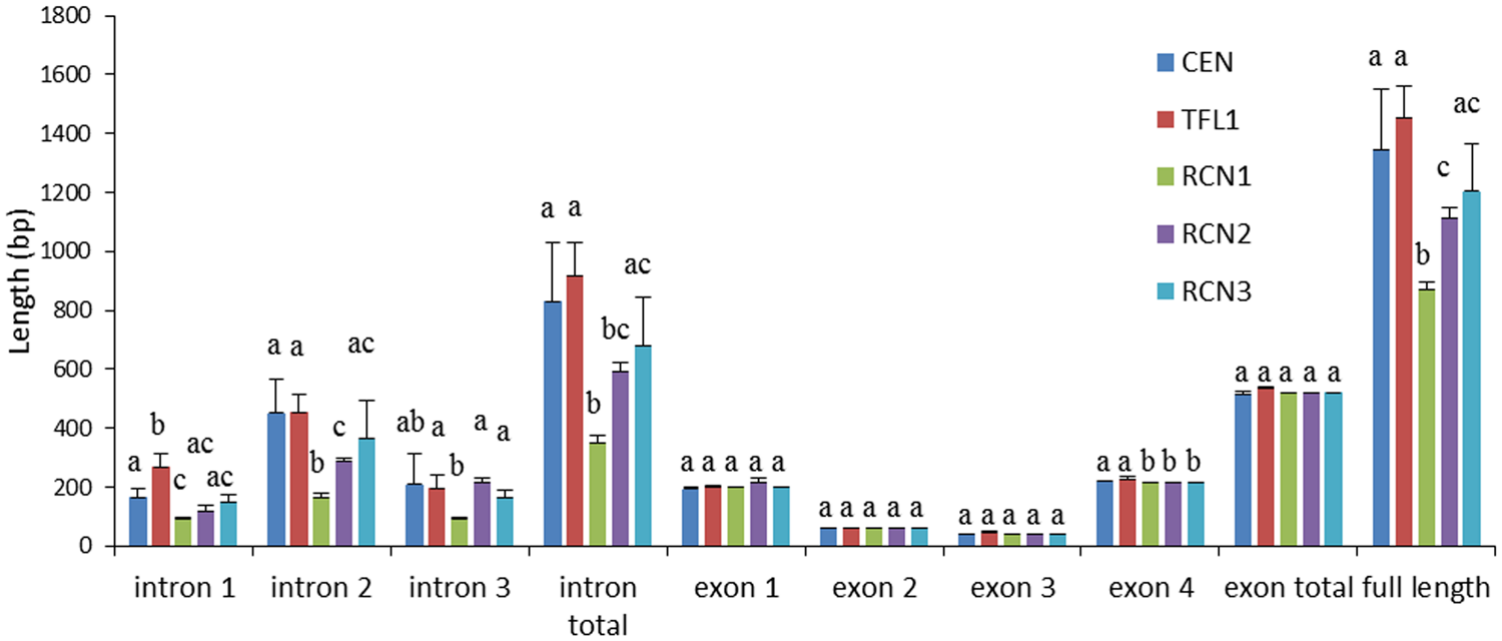
Length polymorphisms of eudicot and monocot *TFL1/CEN/RCN* paralogs. Error bars represent one standard error. Different colors represent different *TFL1/CEN/RCN* lineages. Introns have greater length variation than exons, and the introns of monocot *RCN1* are significantly shorter than other paralogs. Levels not connected by the same letter are significantly different based on Student’s t test.

**Figure 3:**
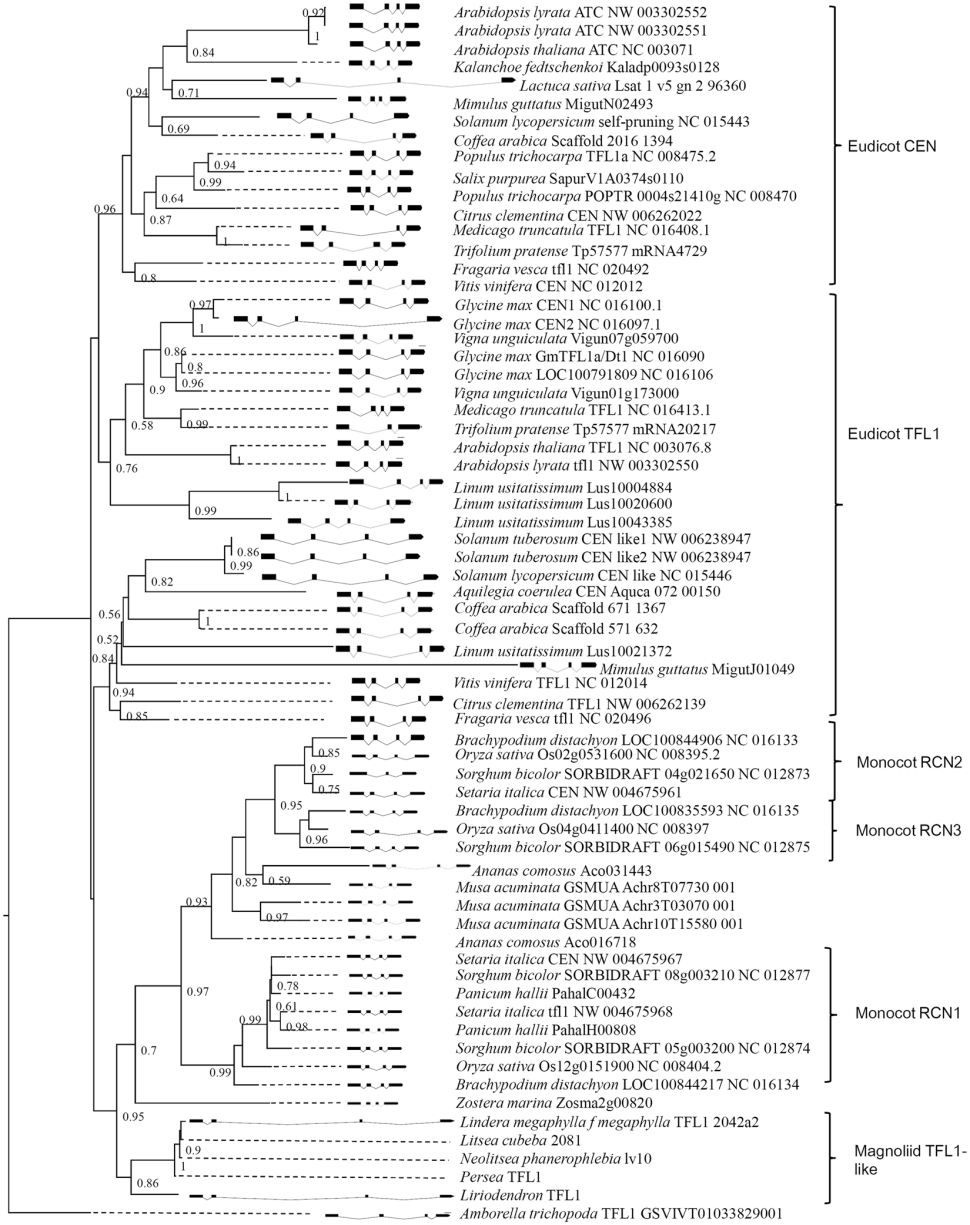
Maximum likelihood tree and the exon-intron structure of angiosperm *TFL1/CEN/RCN* paralogs. Values of the nodes are bootstrapping supports for grouping. The bold boxes indicate the exon while the curves indicate the intron.

All sequences were confirmed as *TFL1*-like by the presence of histidine at the 92^nd^ amino acid position (corresponding position at the 88^th^ site of *Arabidopsis*)^25^. Only one copy for each Magnoliid species was obtained after amplification, and this result is consistent with only one *TFL1/CEN/RCNs* member in EST-library of basal angiosperm database (accession number: gnl|Liriodendron|b4_c119764, Ancestral Angiosperm Genome Project, http://ancangio.uga.edu/index.php). The sequences amplified from Magnoliid shown the best hit to *TFL1/CEN/RCNs* family (*Nelumbo nucifera CEN*-like protein 2, E-value: 5e^−99^–2e^−92^). Exon lengths of eudicot *TFL1* and *CEN*, monocot *RCN1*, *RCN2*, and *RCN*3, and basal angiosperm (magnoliids + *Amborella*) *TFL1*-like gene range 519–609 bps, 447–531 bps, 522 bps, 522 bps, 522 bps and 516–522 bps, respectively. The length of introns from *TFL1*, *CEN*, *RCN1*, *RCN2*, *RCN3*, and basal angiosperm *TFL1*-like genes are 496–2048 bps, 320–3273 bps, 312–384 bps, 509–1007 bps, 510–643 bps, and 1444–3539 bps, respectively. Exon lengths were found to be constant and there were no significant differences between paralogs, with the exception of exon 4 between eudicots and monocots (Fig. 2). In contrast, the intron lengths were highly variable, and the monocot *RCN1* was found to have relatively short but constant intron lengths compared with other paralogs. Furthermore, monocot *RCNs* had a higher number of intron length polymorphisms than eudicot *TFL1/CEN* (Fig. 2). Although only two *TFL1*-like full sequences were obtained from magnoliids, the synapomorphy of the long intron lengths of *TFL1*-like genes in Lauraceae and Magnoliaceae were confirmed by PCR (Additional file 1: Fig. S1). Analysis revealed that the monocot *Sorghum bicolor* has lost intron 1, and that exon 2 has merged with exon 1, and this sequence is removed when estimating the exon/intron length variation. The exon/intron structures and lengths are shown in Fig. 3 and Additional file 1: Table S2.

### Correlation between intron lengths and conserved motifs

**Figure 4:**
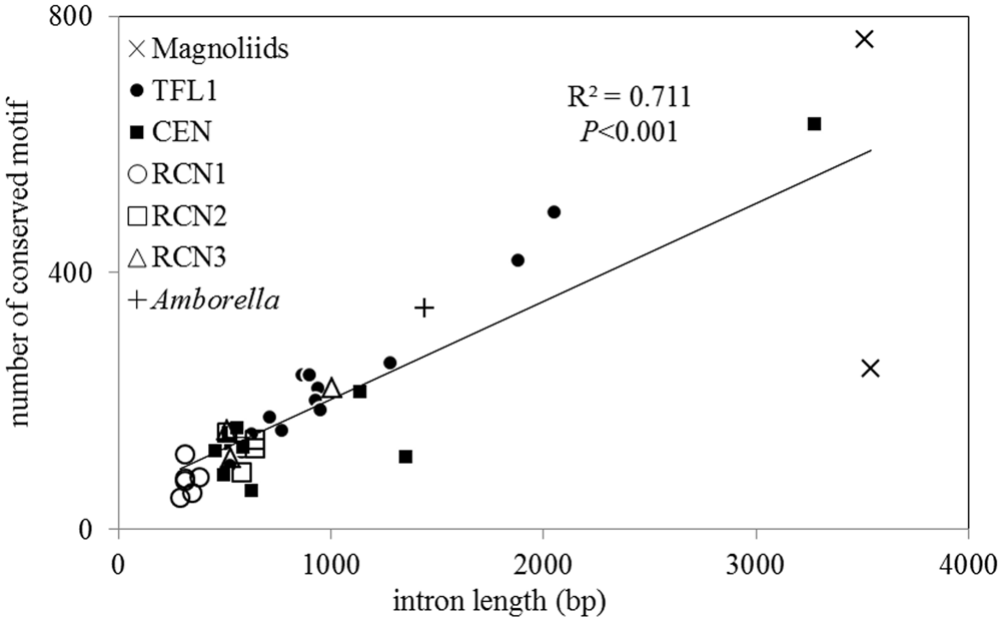
Significant positive correlation between the number of *cis*-acting elements and intron length. The correlation coefficient (R^2^) and significance of the correlation coefficient (*P*) were calculated. Intron length variations are listed in Additional file 1: Table S2. Types and locations of the putative *cis*-acting elements are listed in Additional file 1: Table S3.

Twelve conserved motifs, which are identical to the motifs of putative *cis*-acting elements, were identified in noncoding regions (Additional file 1: Table S3), and the four-base motifs CAAT box and WRKY were abundant and both presence frequencies (0.0054 and 0.0064, respectively) are higher than those predicted by random occurrence (>1/256, *p* = 0.0245 and 0.0001, respectively) (Additional file 1: Table S3). The *TFL1*-like gene from magnoliids was found to have longer introns and more abundant *cis-*element to motifs. A strong and significant positive correlation between the number of *cis-*element motifs and intron length were found (*R* ^2^ = 0.711, *p* < 0.0001, Fig. 4), suggesting that noncoding regions in *TFL1/CEN/RCNs* paralogs are relevant to intron length.

### Phylogenetic tree of angiosperm *TFL1/CEN/RCNs* paralogs

The phylogenetic tree of angiosperm *TFL1/CEN/RCNs* paralogs was reconstructed using amino acid sequences (Fig. 3) and was inconsistent with the hypothetical tree (Fig. 1 and Additional file 1: Fig. S2). The magnoliid *TFL1*-like gene was misgrouped with monocot *RCNs* (Fig. 3). The misgrouping for monocot and magnoliid paralogs was also revealed in the bush-like tree topology for basal lineages by Bayesian inference (Additional file 1: Fig. S3). The misgrouping of Magnoliid with monocot or eudicot is common in phylogenetic analysis using certain genes, which is probably due to combination of the relatively old age of these taxa and long branches attraction^40–42^. In contrast to the unresolved topology of basal lineages of eudicot and magnoliid paralogs, the monocot *RCN* paralogs were well grouped, with relatively high bootstrap supports in the ML and Bayesian trees (Fig. 3 and Additional file 1: Fig. S3). Furthermore, *RCN2* and *RCN3* are grouped together in both ML and Bayesian analyses (Fig. 3), implying that the duplication sequence in monocots is *RCN1* and *RCN2*/*3* followed by *RCN2* and *RCN3*.

### Positive selection analyses

To examine the effect of selective pressures on the angiosperm *TFL1/CEN/RCNs* paralogs, the ratio (*ω*) of missense (*Ka*) to silent mutation rates (*Ks*), an indicator of natural selection, was estimated. Likelihood ratio analysis revealed that the free ratio model was a better fit than the constant ratio model (2∆L = 97.8117, df = 61, *P* = 0.0004), suggesting a strong and pervasive purifying selection on angiosperm *TFL1/CEN/RCNs* paralogs (Fig. 1). To examine the grouping of magnoliid *TFL1*-like gene with eudicot *TFL1/CEN* and monocot *RCNs* (Table 1), the two and three ratio models were performed, and showed relaxation of selective constraints (*ω*_0_ < *ω*_1_, *ω*_2_ < 1) for eudicot *TFL1*, *CEN*, monocot *RCN2* and *RCN3*, and magnoliid *TFL1*-like gene, but strong purifying selection (*ω*_0_ > *ω*_1_) for the monocot *RCN1* (Table 2). The two ratio model was a better fit for the evolution of monocot, eudicot and magnoliids *TFL1/TFL1*-like paralogs than three ratio models. This suggests that the grouping of eudicot and magnoliid *TFL1/TFL1*-like paralogs is a consequence of functional constraint and that both paralogs suffered different selective pressures for magnoliids *TFL1/TFL1*-like paralogs (Table 2).

**Table 1:** Hypotheses and the corresponding scenarios for the grouping of eudicot *TFL1* and magnoliid *TFL1*-like genes

Hypotheses	Scenarios
1.*ω*_1_ = *ω*_2_ ≤ 1	Functional constraint hypothesis
2.*ω*_1_ = *ω*_2_ > 1	Synchronous selection hypothesis
3.*ω*_1_ ≠ *ω*_2_	Phylogenetic convergence driven by different selective pressures
3.1.*ω*_1_ > 1, *ω*_2_ > 1	Different strengths of positive selection on eudicot *TFL1* and magnoliid *TFL1*-like
3.2.*ω*_1_ > 1, *ω*_2_ ≤ 1	Positive selection drives the convergence of eudicot *TFL1* into magnoliid *TFL1*-like
3.3.*ω*_2_ > 1, *ω*_1_ ≤ 1	Positive selection drives the convergence of magnoliid *TFL1*-like into eudicot *TFL1*
3.4.*ω*_0_ < *ω*_1_ ≤ 1	Relaxation of selective constraints for eudicot *TFL1*
3.5.*ω*_0_ < *ω*_2_ ≤ 1	Relaxation of selective constraints for magnoliid *TFL1*-like
3.6.*ω*_1_ ≤ *ω*_0_ ≤ 1	Purifying selection on eudicot *TFL1*
3.7.*ω*_2_ ≤ *ω*_0_ ≤ 1	Purifying selection on magnoliid *TFL1*-like

*ω*_1_, *ω*_2_, and *ω*_0_ are the *Ka*/*Ks* ratio of the branches of eudicot *TFL1*, magnoliid *TFL1*-like, and the other lineages (backgrounds), respectively. The *ω*_1_ was also set for the eudicot *CEN* and monocot *RCN1*~*3* for testing the same hypotheses.

**Table 2:** Summary of the *ω* estimation and likelihood ratio test (2ΔL) between two-ratio (*ω*_0_ ≠ *ω*_1_ = *ω*_2_) and three-ratio (*ω*_0_ ≠ *ω*_1_ ≠ *ω*_2_) models.

Hypothesis	np	lnL	2ΔL	*p*	*ω*	Supporting hypothesis in Table 1
*TFL1* vs. magnoliids						1. Functional constraint hypothesis
*ω*_1_ = *ω*_2_	135	−12594.3259			*ω*_0_ = 0.1044, *ω*_1_ = *ω*_2_ = 0.13221	
*ω*_1_ ≠ *ω*_2_	136	−12593.9631	0.7256	0.3258	*ω*_0_ = 0.1021, *ω*_1_ = 0.1361, *ω*_2_ = 0.1678	
*CEN* vs. magnoliids						1. Functional constraint hypothesis
*ω*_1_ = *ω*_2_	135	−12597.2165			*ω*_0_ = 0.1173, *ω*_1_ = *ω*_2_ = 0.1072	
*ω*_1_ ≠ *ω*_2_	136	−12597.2164	0.0002	0.9887	*ω*_0_ = 0.1054, *ω*_1_ = 0.1193, *ω*_2_ = 0.1674	
*RCN1* vs. magnoliids						1. Functional constraint hypothesis
*ω*_1_ = *ω*_2_	135	−12596.0957			*ω*_0_ = 0.1170, *ω*_1_ = *ω*_2_ = 0.0891	
*ω*_1_ ≠ *ω*_2_	136	−12595.6616	0.8682	0.2774	*ω*_0_ = 0.1140, *ω*_1_ = 0.0841, *ω*_2_ = 0.1665	
*RCN2* vs. magnoliids						1. Functional constraint hypothesis
*ω*_1_ = *ω*_2_	135	−12597.6561			*ω*_0_ = 0.1137, *ω*_1_ = *ω*_2_ = 0.1164	
*ω*_1_ ≠ *ω*_2_	136	−12597.5681	0.176	0.8708	*ω*_0_ = 0.1137, *ω*_1_ = 0.1078, *ω*_2_ = 0.1240	
*RCN3* vs. magnoliids						1. Functional constraint hypothesis
*ω*_1_ = *ω*_2_	135	−12597.2484			*ω*_0_ = 0.1126, *ω*_1_ = *ω*_2_ = 0.1314	
*ω*_1_ ≠ *ω*_2_	136	−12596.6628	1.1712	0.2052	*ω*_0_ = 0.1126, *ω*_1_ = 0.1079, *ω*_2_ = 0.1535	
Eudicots vs. magnoliids						1. Functional constraint hypothesis
*ω*_1_ = *ω*_2_	135	−12592.2355			*ω*_0_ = 0.0915, *ω*_1_ = *ω*_2_ = 0.1255	
*ω*_1_ ≠ *ω*_2_	136	−12592.0526	0.3658	0.5494	*ω*_0_ = 0.0916, *ω*_1_ = 0.1085, *ω*_2_ = 0.1264	
Monocots vs. magnoliids						1. Functional constraint hypothesis
*ω*_1_ = *ω*_2_	65	−12592.9536			*ω*_0_ = 0.1249, *ω*_1_ = *ω*_2_ = 0.0931	
*ω*_1_ ≠ *ω*_2_	66	−12592.7766	0.354	0.5617	*ω*_0_ = 0.1249, *ω*_1_ = 0.1073, *ω*_2_ = 0.0917	

*ω*_1_, *ω*_2_, and *ω*_0_ are the *Ka*/*Ks* ratio of the branches of the eudicot *TFL1* (or eudicot *CEN*, monocot *RCNs*), magnoliid *TFL1*-like, and background lineages, respectively.

np: number of parameters

*p*: *p*-value obtained from fitted model using *χ* ^2^ test.

### Evolutionary divergence between angiosperm *TFL1/CEN/RCNs* paralogs

**Figure 5:**
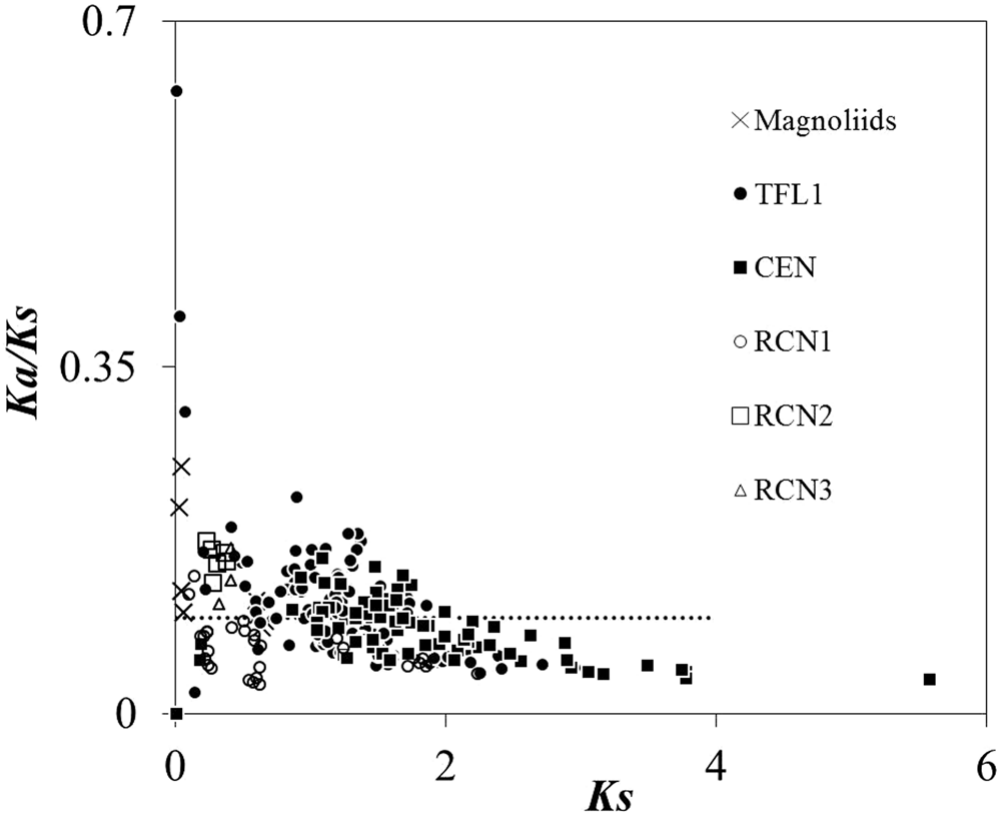
The *Ka/Ks* ratios against *Ks* values of pair comparisons of *TFL1/CEN/RCN* paralogous sequences within clades. The full and open symbols indicate the eudicot and monocot paralogous clades, respectively. The horizontal dotted line indicates the average *Ka/Ks* ratio (=0.0791) of all angiosperm *TFL1/CEN/RCN* paralogous sequences.

The pairwise *Ka/Ks* ratio was calculated and plotted against *Ks* to reveal patterns of selection through time. No pairwise *Ka/Ks* > 1 were obtained suggesting that no positive divergent selection occurred between paralogs. Eudicot *TFL1* and *CEN* were mostly distributed in the quadrant *Ka/Ks* < 1 and *Ks* > 1, indicating long-term purifying selection. The monocot *RCNs* and magnoliids *TFL1*-like genes were distributed in the quadrant *Ka/Ks* < 1 and *Ks* < 1. We divided this quadrant into two classes: (1) *Ka/Ks* > 0.097 (average *Ka/Ks*), suggesting the relaxation of selective constraints. This quadrant comprises the magnoliid *TFL1*-like and the monocot *RCN2* and *RCN3*; and (2) *Ka/Ks* < 0.097, suggesting strong selective constraints or recent purifying selection, which comprised the monocot *RCN1* (Fig. 5). This inference is consistent with the results of tests for selection hypotheses (Table 2).

**Figure 6:**
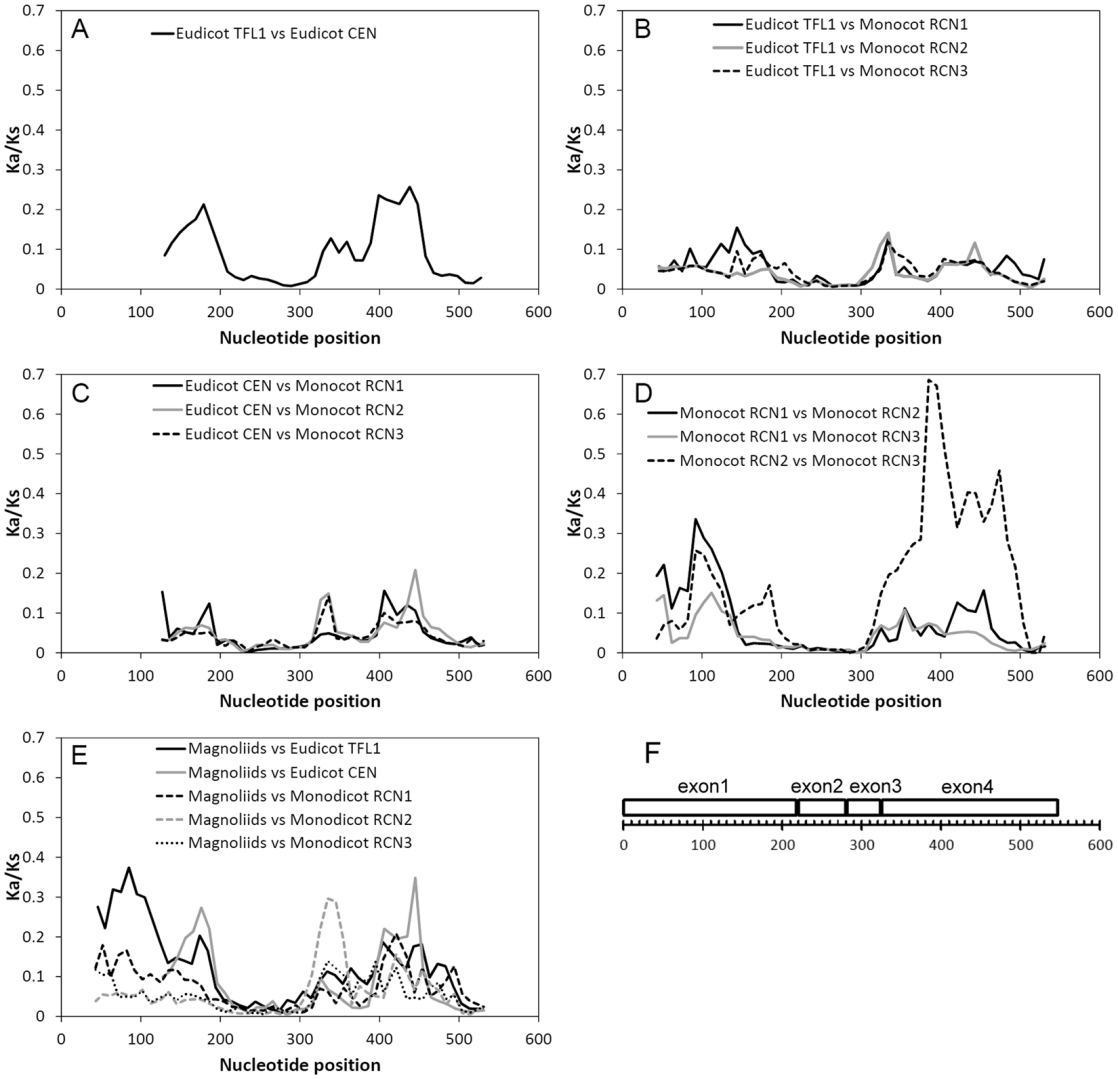
*Ka/Ks* sliding windows of 50 nucleotides with a 10-bp step size between angiosperm *TFL1/CEN/RCN* paralogs. Comparisons (**A**) between eudicot *TFL1/CEN* paralogs, (**B**) between eudicot *TFL1* and monocot *RCNs*, (**C**) between eudicot *CEN* and monocot *RCNs*, (**D**) between monocot *RCN* paralogs, and (**E**) between magnoliid *TFL1*-like, and eudicot and monocot *TFL1/CEN* paralogs. (**F**) The corresponding alignment positions of exons, revealing selective constraints on exon 2 and exon 3. The midposition of windows were listed in base pair (bp).

The sliding window analysis showed pairwise *Ka/Ks* < 1 in all regions among paralogs, with the greatest evolutionary divergence in exon 1 and exon 4 (Fig. 6). Relatively conserved regions in exon 2 and exon 3 indicate that these regions were subject to strong selective constraints. Relatively high *Ka/Ks* at exon 4 between the recently divergent monocot *RCN2* and *RCN3*, indicate that this was subject to low selective pressures of constraining amino acid changes between *RCN2* and *RCN3* (Fig. 6D). Small *Ka/Ks* ratios between eudicot and monocot paralogs indicate functional conservatism divergence (Fig. 6B and C). Magnoliid *TFL1*-like gene was found to have a highly divergent exon 1 and exon 4 compared with the other paralogs (about 100^th^ bp in *TFL1*, 180^th^ and 450^th^ bp in *CEN*, 340^th^ bp in *RCN2*). This suggests that this gene was subject to different selective pressures than the eudicot and monocot paralogs, while the conservation of exon 2 and exon 3 suggests long-term and pervasive functional constraints on these genetic regions (Fig. 6E).

### Radical functional divergence between angiosperm *TFL1/CEN/RCNs* paralogs

An *in silico* analysis of radical amino acid changes between duplicated genes was conducted for testing the functional divergence of *TFL1/CEN/RCNs* paralogs. Nonsignificant radical functional divergence, as estimated by the type-II functional divergence index (θ_II_)^43^, was found between angiosperm *TFL1/CEN/RCNs* paralogs (Table 3). The proportion of fixed radical change between paralogs (F_00,R_) was zero between eudicot *TFL1/CEN* and paralogs of monocots and magnoliids, but more or less between paralogs between monocot *RCNs* and magnoliid *TFL1*-like genes (Table 3). This indicates that there is functional conservation of paralogs between eudicots and monocots, and functional specialization between paralogs within the eudicot and monocot species.

**Table 3:** Summary of type-II functional divergence analysis for angiosperm *TFL1/CEN/RCNs/TFL1-*like paralogs.

	θ_II_ ± SE	*p*-value	a_R_/π_R_	G_R_	G_C_	F_00,N_	F_00,R_	F_00,C_
*TFL1/CEN*	−0.181 ± 0.089	0.156	0.683	0.548	0.452	0.306	0	0
*TFL1/RCN1*	0.060 ± 0.059	0.212	−0.544	0.655	0.345	0.376	0	0
*TFL1/RCN2&3*	−0.132 ± 0.084	0.235	−0.227	0.591	0.409	0.316	0.003	0
*TFL1/*magnoliids	—	1	—	1	0	0	0	0
*CEN/RCN1*	−0.111 ± 0.068	0.245	−0.738	0.608	0.392	0.503	0.01	0.008
*CEN/RCN2&3*	−0.142 ± 0.074	0.253	−0.072	0.570	0.430	0.376	0	0
*CEN/*magnoliids	—	1	—	1	0	0	0	0
*RCN1/RCN2&3*	−0.058 ± 0.053	0.177	−0.184	0.457	0.543	0.503	0.010	0.006
*RCN1/*magnoliids	—	1	—	1	0	0	0	0
*RCN2&3/*magnoliids	—	1	-	1	0	0	0	0

θ_II_, coefficient of type-II functional divergence (SE: standard error); *p*-value, significance test based on Z-score test to test the hypothesis of deviation of θ_II_ from zero; a_R_/π_R_: the ratio of radical change under functional divergence versus nonfunctional divergence; G_R_ and G_C_, proportion of radical change and conserved change, respectively; F_00,N_, F_00,R_, and F_00,C_, proportion of none change, radical change, and conserved change of amino acids between clusters but no change within clusters, respectively.

## Discussion

### Exon length conservation and intron length variability

Exon length conservation implies constraints of gene functions among organisms^34,44^. Eudicot and monocot *TFL1/CEN/RCNs* are functionally conserved and the inflorescence architecture was determined by comparison with the model organisms *Arabidopsis* and rice^45^. Highly variable intron lengths and sequences of angiosperm *CEN/RCNs/TFL1*-like genes suggest absence of constraining reproductive function from noncoding regions. It is not known whether intron fragments have been gained or lost through evolution, due to poor or failed alignment in introns. However, we suspect that there was a gradual deletion throughout intron evolution because, generally, there are longer introns in basal angiosperms than in both eudicots and monocots (Fig. 3 and Additional file 1: Fig. S1). A deletion of this type in introns could be the result of recombination^46^ and may have contributed to the divergence and functional differentiation in this family of genes^47^. Intron lengths are positively correlated with the number of conserved motifs, which are identical to the putative transcription factor binding sites (*R* ^2^ = 0.711, *P* < 0.0001, Fig. 4). Furthermore, certain motifs in intron may stimulate gene expression^48^. Long introns with more conserved motifs could have a complicated folding structure, as well as alternative splicing sites that affect transcription, particularly for the basal angiosperms (such as Lauraceae, Magnoliaceae, and *Amborella*). Alternative splicing in *TFL1/CEN* paralogs was reported to influence terminal flowering and flowering time^49^. Formation of gene loops is also relevant to the activation or maintenance of *Arabidopsis TFL1* expression^45^. Therefore, gene lengths are hypothesised to be a key factor affecting the expression efficiency of *TFL1* orthologs.

*TFL1* is targeted by several MADS-box genes, which have different functions during floral transition, and they coordinate the timing of flowering and floral development with *TFL1* ^45^, indicating that the *TFL1* orthologs could have several protein binding sites. In addition, both AP1 and LFY can bind to the *TFL1* locus and directly suppress *TFL1* expression^50–52^. Suppression of *TFL1* in inflorescence branching regulation by MADS-box genes also affects *LFY* and *AP1* expression^45^. No AP1 binding sites (CArG box) or LFY binding sites were found in either eudicot *TFL1* and monocot *RCN1*, but were present in basal angiosperms (Additional file 1: Table S3). This might suggest the functional relevance of long introns in the *TFL1*-like gene in basal angiosperms. In contrast, the short introns of eudicot and monocot paralogs could reflect their low expression^33^, which may facilitate the retention of duplicated genes and the conservation of their ancestral functions^53^.

### Pervasive purifying selection and relaxation of selective constraints on eudicot and monocot *TFL1/CEN/RCNs* paralogs

It was suggested that the functions of angiosperm flowering development genes, have been conserved under selective constraint in eudicots and monocots^34,45,54^. Strong purifying selection of the *TFL1* paralog with an average *ω* = 0.097 was inferred based on site-model analysis^34^, which is similar to the average pairwise *Ka/Ks* = 0.0791 estimated in our analysis (the horizontal dotted line in Fig. 5), and lower than the average *ω* of other floral-regulatory paralogs (*SEP1* vs. *SEP2* and *SHP1* vs. *SHP2*, both *ω* = 0.16^38^). Nonsignificant radical functional divergence (θ_II_) between paralogs supports the functional constraint hypothesis for angiosperm *TFL1/CEN/RCNs* paralogs (Table 3). However, in the phylogenetic analysis (Fig. 3 and Additional file 1: Fig. S3), low bootstrap values or posterior probabilities of basal lineages of the magnoliid *TFL1*-like and eudicot *TFL1*/*CEN* paralogs suggest a lack of fixed differences between paralogs. This indicates that multiple common polymorphisms are shared between clades or within-clade evolutionary constraints.

**Figure 7:**
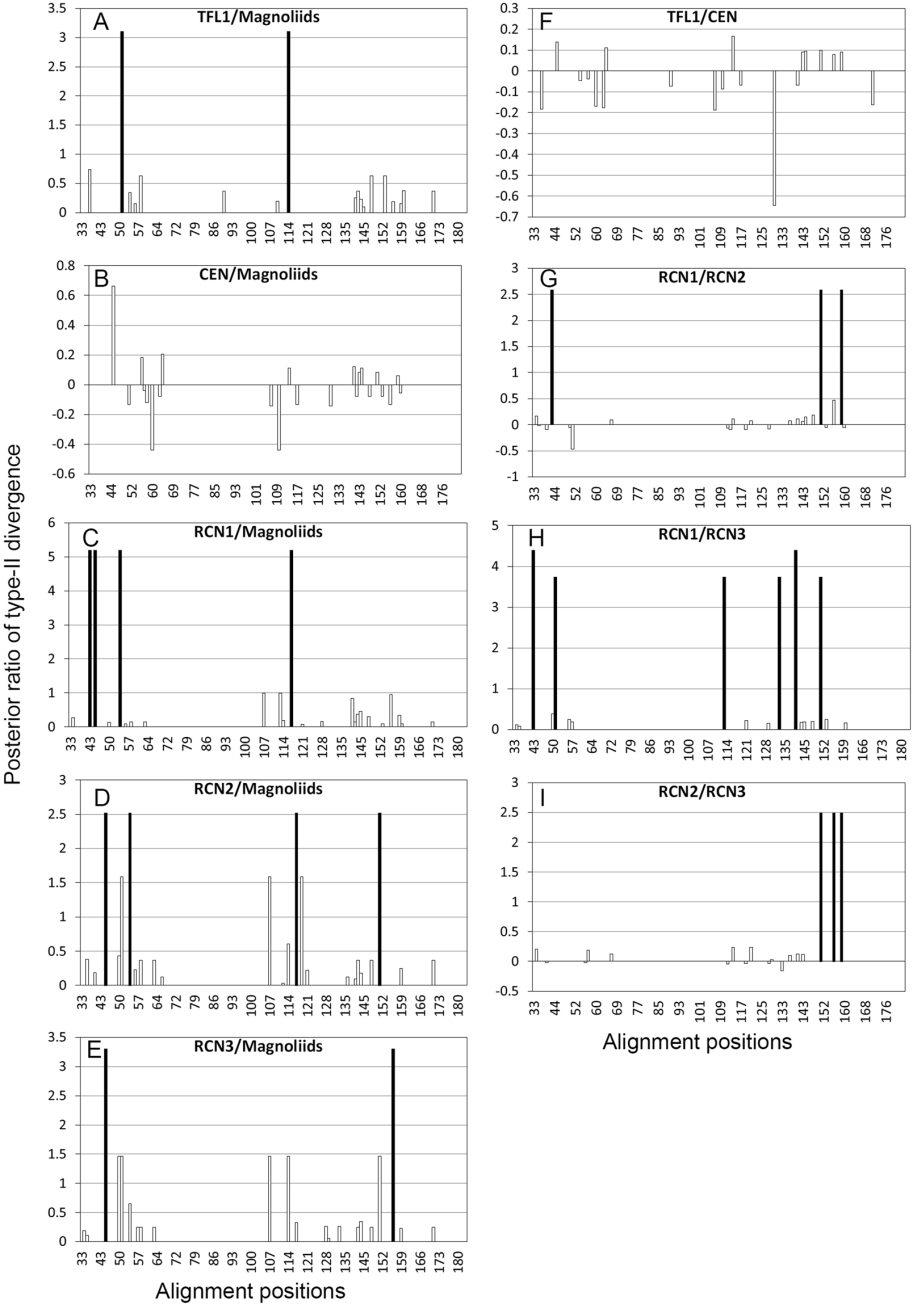
Site-specific profile of type II functional divergence between angiosperm *TFL1/CEN/RCN* paralogs. Only the comparisons between the magnoliid *TFL1*-like and other paralogs, between eudicot *TFL1* and *CEN*, and between monocot *RCN1*, *RCN2*, and *RCN3* are shown. The full bars indicate the critical posterior ratio with a posterior probability >0.7.

The pairwise *Ka/Ks* ratio and the sliding window analysis suggest that there were long-term selective constraints on eudicot *TFL1* and *CEN* (Fig. 5), particularly on exon 2 and exon 3 of all angiosperm *TFL1/CEN/RCNs* paralogs (Fig. 6). Exon 2 and exon 3 are activator regions (ligand-binding site) of *TFL1* ^25,55^, and are highly conserved with no amino acid changes (Fig. 7). However, relatively higher pairwise *Ka/Ks* within monocot and magnoliid paralogs suggests that constraints were relaxed, particularly in exon 1 and exon 4 (Figs. 5 and 6), which is also supported by the analysis of site-specific radical functional change between paralogs of both monocots and magnoliids (Fig. 7). Residues 133–151 in exon 4 form an external loop, and this conformation determines the functional specificities of floral regulators^55^. Loss of the hydrogen bond between the external loop (exon 4) and the activator regions (exon 3) may be responsible for the functional conversion of activators of FT to floral repressors of TFL1^55^. One radical change in *RCN1*/*RCN2*, *RCN2*/*RCN3*, *RCN2*/magnoliid *TFL1*-like, and three radical changes in *RCN1*/*RCN3* within the external loop were estimated (Fig. 7), suggesting that the paralogous divergence occurred by relaxation of selective constraints, particularly between the monocot *RCNs*.

Limited fixed radical differences (F_00,R_) could suggest the maintenance of ancestral function between paralogs of different taxa and imply that the eudicot *TFL1*/*CEN* and monocot *RCNs* do not fit the neo-functionalisation hypothesis of duplicate genes. Instead, the duplication could be a case of sub-functionalisation due to the relaxation of functional constraints, because one-third to a half frequency of radical change (G_R_) was detected (Table 3). The duplication-degeneration-complementation (DDC)^56^ and escape from adaptive conflict (EAC) models^57^ are commonly used to explain subfunctional divergence of duplicates and retention of duplicates^58^. The main difference between the DDC and EAC models is that the EAC puts more emphasis on positive selection for gene specialisation during or after duplication, while positive selection is not required for DDC^59^. In the case of eudicot *TFL1/CEN* and monocot *RCNs*, all duplicates retained plesiomorphic functionality with slight differences, by relaxation of selective constraints. However, no specific paralogs suffered positive selective pressures, suggesting a more likely evolutionary fit to DDC. The EAC hypothesis therefore, was rejected.

### Relaxation of selective constraints and phylogenetic convergence of magnoliid *TFL1*-like genes

No duplication of *TFL1-*like genes was found in basal angiosperms. The constructed phylogenetic tree showed that the magnoliid *TFL1*-like genes are grouped with eudicot *TFL1/CEN* paralogs (Fig. 3 and Additional file 1: Fig. S3). This may suggest: (1) constraining ancestral functions of the eudicot *TFL1* with the basal-angiosperm *TFL1*-like gene (functional constraints hypothesis), (2) identical selection pressures acted on both eudicot *TFL1* and magnoliid *TFL1*-like genes synchronously (synchronous selection hypothesis), or (3) eudicot *TFL1* and magnoliid *TFL1*-like genes evolved in parallel independently, resulting in phylogenetic convergence (phylogenetic convergence hypothesis). The LRTs for the two ratio and three ratio models showed the functional constraints between magnoliid *TFL1*-like and other paralogs (*ω*_1_ = *ω*_2_ ≤ 1) except the monocot *RCN1* (*ω*_0_ ≠ *ω*_1_ ≠ *ω*_2_) (Table 2). This indicates that (1) eudicot and monocot *TFL1/CEN/RCNs* could share ancestral polymorphisms and functions with *TFL1*-like gene of basal angiosperms, and (2) magnoliid *TFL1*-like and monocot *RCN1* could functionally converge under heterogeneous evolutionary rates. The basic functions of *TFL1/CEN/RCNs* paralogs in magnoliids, eudicots, and monocots do not alter, but there is division of labour by small fractions of neutral or nearly neutral amino acid replacements, which is consistent with the functional divergence analysis (Table 3).

The long-term constrained evolution of floral development genes across divergent species was inferred by comparative analyses of 18 angiosperm species^34^. However, the evolutionary pattern of these genes in basal angiosperms, such as *Amborella*, Lauraceae, and Magnoliaceae, has not yet been investigated. Although the functional constraint hypothesis was supported between magnoliid *TFL1*-like genes and most other paralogs, the *ω* of foreground branches are larger than background lineages (Table 2), supporting the hypothesis of relaxation of constraints for flexing the non-duplicated magnoliid *TFL1-*like genes in shaping floral diversity inferred by both pairwise *Ka/Ks* (Fig. 5) and functional divergence analysis (Table 3). The relaxation of selective constraints was common for duplicated genes at the phase of early duplication that accelerated evolution of duplicated genes to escape from redundancy, while most gene duplicates were stochastically silenced with few survivors subsequently experiencing strong (10-fold efficiency) purifying selection^14^. Here, we provide at least two novel discoveries regarding the evolution of *TFL1*-like genes in basal angiosperms: (1) Lauraceae and Magnoliaceae *TFL1*-like genes are divergent from those of *Amborella* and are phylogenetically similar to the eudicot *TFL1/CEN*; (2) purifying selection prevailed over the magnoliid *TFL1*-like genes as well as the eudicot and monocot paralogs, but the unfixed paralogous radical replacement enabled their differentiation through the relaxation of selective constraints.

## Conclusions

In this work, we inferred evolution and functional divergence of *TFL1/CEN/RCN* among 18 angiosperm species, including basal angiosperm species to elucidate the duplication history of *TFL1/CEN/RCN* genes. We found long-term retention of functionally redundant duplicates *TFL1/CEN/RCNs* in the angiosperm genomes. Based on the results of purifying selection on exon, radical amino acid changes and various intron lengths with *cis*-acting element analysis, the maintenance and conservation of their ancestral function could be explained by duplication-degeneration-complementation model. The ancestral function of *TFL1/CEN/RCNs* might be preserved and divided into each duplicates. Therefore, the strong selection pressure against removing any duplicates may cause the permanent establishment of duplicates during evolution of flowering plants. Consequently, these two duplicates together maintain the conservative mechanism in inflorescence architectures, and expansion of the PEBP gene members may be important factor for driving morphological divergence among angiosperms.

Intron length of *TFL1* paralogs was various. *TFL1* introns of basal angioserpm tend to have longer intron and more predicted *cis*-acting than monocot and eudicot. On the other hands, exon length was conserved with low amino acid substitution rate. These data suggest that strong purifying selection has maintained the relevant functions of *TFL1/CEN/RCNs* paralogs on flowering regulation throughout the evolution of angiosperms, and the shorter introns with radical amino acid changes are important for the retention of paralogous duplicates.

## Methods

### Data collection and phylogenetic tree reconstruction

The full lengths of angiosperm *TFL1/CEN/RCNs* genes were obtained from NCBI GenBank. Organisms without complete paralogs (e.g. only *TFL1* of eudicot and *RCN1* of monocot organisms) were excluded. Due to high similarity among PEBP gene family, many sequences named with *TFL1* or *CEN* are belonged to *FT/BFT/MFT*. For preventing miss-inferring of phylogenetics of *TFL1/CEN*, we only included sequences which were previously identified as *TFL1/CEN* in our subsequent analysis e.g. ref.^7^. The *TFL1* and *CEN* gene sequences from five eudicot species (*Arabidopsis thaliana*, *A. lyrata* [Brassicaceae], *Citrus clementina* [Rutaceae], *Fragaria vesca* [Rosaceae], *Glycine max* [Fabaceae], *Medicago truncatula* [Fabaceae], *Populus trichocarpa* [Salicaceae], *Solanum lycopersicum* [Solanaceae], *Solanum_tuberosum* [Solanaceae], *Vitis vinifera* [Vitaceae], *Linum usitatissimum* [Linaceae], *Kalanchoe fedtschenkoi* [Crassulaceae], *Mimulus guttatus* [Phrymaceae], *Salix purpurea* [Salicaceae], *Trifolium pratense* [Fabaceae], *Vigna unguiculata* [Fabaceae], *Lactuca sativa* [Asteraceae], *Coffea arabica* [Rubiaceae],), and *RCN1*–*3* from four monocot species (*Musa acuminata* [Musaceae], *Ananas comosus* [Bromeliaceae], *Zostera marina* [Zosteraceae], *Oryza sativa*, *Sorghum bicolor*, *Setaria italic*, and *Brachypodium distachyon*, *Panicum hallii* [Poaceae]), and the *TFL1*-like gene from *Amborella trichopoda* (Amborellaceae) were obtained from GenBank. We also amplified complete *TFL1*-like sequences from two basal angiosperm species *Lindear megaphylla* (Lauraceae) and *Liriodendron* sp. (Magnoliaceae) using primers (MaLaTFL1-F1: 5′-ATGGCAAGAATGTTAGAGC-3′; MaLaTFL1-R1: 5′-CAACGTCTCCTNGCAGCTG-3′). Intron positions were rechecked based on the GT-AG rule. Exon-intron structures were drawn by Exon-Intron Graphic Maker (http://wormweb.org/exonintron). Exons of *Litsea cubeba*, *Neolitsea phanerophlebia*, *Persea* sp., and *Michelia compressa* (GenBank accession number: KY933631- KY933636) were also sequenced for coding region analyses. The identification of the exon sequences were conducted using BLAST. Sequences without best hit to *TFL1/CEN/RCNs* were discarded (eg. *FT/BFT/MFT*). The phylogenetic tree of *TFL1/CEN/RCNs* was reconstructed by exons using the Maximum likelihood method with the GTR+G model, gamma distribution (α = 0.46) for substitution rate among sites using PhyML 3.0^60^. The tree bisection and reconnection (TBR) was adopted for tree rearrangement and fast bootstrap method aLRT was adopted for branch supports.

### Conserved motifs in introns

Conserved motifs in introns were found by searching the database of plant *cis*-acting regulatory DNA elements, NEW PLACE^61^. From 212 types of predicted motifs like *cis*-acting elements, 12 putative functional *cis*-acting elements that have been reported to regulate the expression of *TFL1/CEN/RCNs* paralogs were identified (Additional file 1: Table S1)^49,62–65^ and the number of these putative *cis*-acting elements were calculated. Correlation between the number of *cis*-acting elements and the total intron length (*i.e*. intron1 + intron2 + intron3) was estimated.

### Detection of positive selection on angiosperm *TFL1/CEN/RCNs* paralogs

To examine the effect of selective pressures, the *ω* ratio, which can be used for testing the gene neutrality hypothesis *Ka*/*Ks* (*ω*) = 1, was estimated by maximum likelihood approaches implemented in PAML 4.7^66^. First, the *ω* under the free-ratio model was estimated, which allows varied *ω* on every branch. The likelihood ratio test (LRT) that calculates the 2× differences of log likelihood between constant-rate model and other evolutionary hypotheses (2ΔL) were used for evaluating the better fitted selective hypothesis by *χ* ^2^ test. Because *TFL1*-like genes in magnoliids were grouped with eudicot *TFL1/CEN* clades (Fig. 1), we hypothesized that (1) functional constraints between magnoliid *TFL1*-like and eudicot *TFL1/CEN* and (2) phylogenetic convergence was tested. To test these hypotheses, we estimated the *ω* and evaluated the goodness-of-fit of two-ratio model (*ω*_0_ ≠ *ω*_1_ = *ω*_2_) and three-ratio model (*ω*_0_ ≠ *ω*_1_ ≠ *ω*_2_) by LRT. *ω*_0_ are the *Ka/Ks* ratio of background branches; *ω*_1_ and *ω*_2_ are *Ka/Ks* of foreground branches that allowed *ω* > 1, where *ω*_1_ are the *ω* of eudicot *TFL1/CEN* or monocot *RCNs* paralogs and *ω*_2_ are the *ω* of magnoliid *TFL1*-like genes. Detailed hypotheses and selection scenarios are listed in Table 1.

In addition, pairwise *Ka/Ks* comparisons between angiosperm *TFL1/CEN/RCNs* paralogs were calculated to examine the evolutionary divergence and represented by (1) the *Ka/Ks* against *Ks* plot and (2) sliding window analysis by DnaSP 5.0^67^. The *Ka/Ks* against *Ks* plot helps to determine the degrees and relative times of paralogous divergence and the sliding windows provide details for clarifying the divergent regions from the regions under selective constraints.

### Functional divergence between angiosperm *TFL1/CEN/RCNs* paralogs

Functional divergence between paralogs caused by radical amino acid changes was assessed by the type-II divergence function implemented in DIVERGE 3.0^43^ with 500 bootstrap replications. Substitutions between amino acids with different radical biochemical properties (charge positive/negative, hydrophilic/hydrophobic) are classified as a radical change, and all others are classified as a conserved change. The *Z*-test was conducted to test the deviation of coefficients of type II functional divergence (θ_II_) from zero, and value of θ_II_ greater than zero implied radical shifts in amino acid physiochemical properties after duplication. The fold of radical change related to non-functional change was calculated using the ratio of radical change under functional divergence versus nonfunctional divergence (aR/πR). The proportion of such as fixed radical change, conserved change and none change sites were also calculated. Site-specific estimation of posterior probability of radical changes was performed to assess the probable regions and shifts of biochemical properties between paralogous groups.

### Availability of data and materials

The sequences have been submitted to GenBank with the accession number KY933631-KY933636.

## Electronic supplementary material

Supplementary  Information
