# Supplementary material for: Functional divergence and intron variability during evolution of angiosperm TERMINAL FLOWER1 (TFL1) genes
Source: Sci Rep. 2017 Nov 1;7:14830. doi: 10.1038/s41598-017-13645-0 (PMC5666015; doi:10.1038/s41598-017-13645-0)
Supplement: Supplementary file 1 — Supplementary Information [file 41598_2017_13645_MOESM1_ESM.pdf]

Functional divergence and intron variability during evolution of angiosperm *TERMINAL FLOWER1* (*TFL1*) genes

Jian Gao[1], Bing-Hong Huang[2], Yu-Ting Wan[2], JenYu Chang[3], Jun-Qing Li[1], Pei-Chun Liao[2,*]

[1] College of Forestry, Beijing Forestry University, No.35, Tsinghua East Rd., Haidian Dist., Beijing 100083, People's Republic of China

[2] Department of Life Science, National Taiwan Normal University, No.88, Sec. 4, Tingjhou Rd., Wunshan Dist., Taipei 116, Taiwan, Republic of China

[3] Department of Horticulture, Chiayi Agricultural Experiment Branch, Taiwan Agricultural Research Institute,No.1, Nung-Kai-Chang, Lutsao township, Chiayi 611, Taiwan, Republic of China

Email address:

Jian Gao: gaojian5688@163.com

Bing-Hong Huang: sohjiro4321@yahoo.com.tw

Yu-Ting Wan: myet556610@gmail.com

JenYu Chang: ychang@dns.caes.gov.tw

Jun-Qing Li: lijq@bjfu.edu.cn

Pei-Chun Liao: pcliao@ntnu.edu.tw

* Corresponding Author: Dr. Pei-Chun Liao

ORCID: orcid.org/0000-0003-1733-5504

Email: pcliao@ntnu.edu.tw

Tel: +886-2-77346330

Fax: +886-2-29312904

Table S1 List of conserved motifs with identical sequences of putative *cis*-acting elements found in the introns of angiosperm *TFL1/CEN/RCNs* paralogs

Cis-acting element	Motif	Putative function	Reference
TATA box	TATAAAT	Core promoter element approximately 30 bp of transcription start	(Fu et al., 2014)
ABRE	TACGTG	*cis*-Acting element involved in the abscisic acid responsiveness	(Fu et al., 2014)
AE box	AGAAACAA	Part of a module for light response	(Fu et al., 2014)
ARE	TGGTTT	*cis*-Acting regulatory element essential for anaerobic induction	(Fu et al., 2014)
Box I	TTTCAAA	Light responsive element	(Fu et al., 2014)
CAAT box	CAAT/CAATT	*cis*-Acting element in promoter and enhancer regions	(Fu et al., 2014)
G box	CACGTA	*cis*-Acting regulatory element involved in light responsiveness	(Fu et al., 2014)
MBS	TAACTG	MYB binding site involved in drought-inducibility	(Fu et al., 2014)
ACG box	AACGTT	Unknown	(Tsaftaris et al., 2012)
CCAAT box	CCAAT	Unknown	(Tsaftaris et al., 2012)
CArG box	$CC[A/T]_6GG$	AP1 binding	(West et al., 1997)
LFY binding site	CCAGTG	LEAFY binding	(Benlloch et al., 2011)
WRKY	TGAC	Transcriptional reprogramming regulation; regulate seed germination	(Randoux et al., 2012)

Table S2 Exon and intron lengths of angiosperm *TFL1/CEN/RCNs* paralogs

	Paralog	Intron 1	Intron 2	Intron 3	Intron total	Exon 1	Exon 2	Exon 3	Exon 4	Exon total	Full length
Lindera megaphylla f megaphylla TFL1 2042a2	Magnoliids	137	2187	1190	3514	201	62	41	218	522	4036
Liriodendron TFL1	Magnoliids	141	2292	1106	3539	201	62	41	218	522	4061
Arabidopsis lyrata ATC NW 003302551	*CEN*	326	119	92	537	204	62	41	221	528	1065
Arabidopsis lyrata ATC NW 003302552	*CEN*	326	119	92	537	123	62	41	221	447	984
Arabidopsis thaliana ATC NC 003071.7	*CEN*	353	118	94	565	204	62	41	221	528	1093
Citrus clementina CEN NW 006262022	*CEN*	92	393	101	586	204	62	41	221	528	1114
Coffea arabica Scaffold 2016 1394	*CEN*	121	906	108	1135	201	62	41	218	522	1657
Fragaria vesca tfl1 2	*CEN*	88	442	95	625	210	62	41	218	531	1156
Fragaria vesca tfl1 NC 020492	*CEN*	81	146	93	320	201	62	41	218	522	842
Kalanchoe fedtschenkoi Kaladp0093s0128 exon	*CEN*	130	195	132	457	204	62	41	221	528	985
Lactuca sativa Lsat 1 v5 gn 2 96360	*CEN*	194	1509	1570	3273	192	62	41	218	513	3786
Medicago truncatula TFL1 NC 016408.1	*CEN*	136	1102	111	1349	198	62	41	221	522	1871
Mimulus guttatus MigutN02493	*CEN*	133	267	117	517	183	62	41	227	513	1030
Salix purpurea SapurV1A0374s0110	*CEN*	151	206	138	495	201	62	41	218	522	1017
Vigna unguiculata Vigun07g059700	*CEN*	136	331	90	557	201	62	41	218	522	1079
Vitis vinifera CEN	*CEN*	83	467	107	657	201	62	41	218	522	1179
Aquilegia coerulea CEN Aquca 072 00150	*TFL1*	104	651	109	864	201	62	41	221	525	1389
Arabidopsis lyrata tfl1 NW 003302550	*TFL1*	226	186	84	496	210	62	41	221	534	1030
Arabidopsis thaliana TFL1 NC 003076.8	*TFL1*	210	206	87	503	210	62	41	221	534	1037
Citrus clementina TFL1 NW 006262139	*TFL1*	122	673	140	935	201	62	41	218	522	1457
Coffea arabica Scaffold 571 632	*TFL1*	113	602	213	928	198	62	41	218	519	1447
Coffea arabica Scaffold 671 1367	*TFL1*	113	573	213	899	198	62	41	218	519	1418
Fragaria vesca tfl1 NC 020496	*TFL1*	88	442	95	625	198	62	41	218	519	1144
Linum usitatissimum Lus10004884 exon	*TFL1*	532	309	105	946	225	62	41	224	552	1498
Linum usitatissimum Lus10020600	*TFL1*	378	279	620	1277	198	62	41	227	528	1805

Linum usitatissimum Lus10043385	*TFL1*	95	446	38	579	201	62	41	239	543	1122
Medicago truncatula TFL1 NC 016413.1	*TFL1*	330	94	100	524	201	62	41	221	525	1049
Mimulus guttatus MigutJ01049	*TFL1*	118	351	115	584	210	62	41	230	543	1127
Solanum lycopersicum CEN	*TFL1*	531	1003	514	2048	199	62	41	217	519	2567
Solanum tuberosum CEN like1	*TFL1*	542	734	601	1877	200	62	44	213	519	2396
Trifolium pratense Tp57577 mRNA20217	*TFL1*	605	105	0	710	201	62		346	609	1319
Trifolium pratense Tp57577 mRNA20217	*TFL1*	241	636	184	1061	201	62	101	221	585	1646
Vigna unguiculata Vigun01g173000	*TFL1*	212	433	122	767	201	62	41	218	522	1289
Ananas comosus Aco016718	monocot	162	280	150	592	100	62	41	244	447	1039
Ananas comosus Aco031443	monocot	94	633	252	979	201	62	41	218	522	1501
Musa acuminata GSMUA Achr10T15580 001	monocot	83	194	279	556	201	62	41	218	522	1078
Musa acuminata GSMUA Achr3T03070 001	monocot	96	159	187	442	201	62	41	218	522	964
Musa acuminata GSMUA Achr8T07730 001	monocot	79	251	76	406	204	62	41	221	528	934
Zostera marina Zosma2g00820	monocot	86	86	87	259	201	62	41	218	522	781
Brachypodium distachyon LOC100844217 NC 016134	*RCN1*	100	163	86	349	201	62	41	218	522	871
Oryza sativa Os12g0151900 NC 008404.2	*RCN1*	72	213	97	382	201	62	41	218	522	904
Panicum hallii PahalH00808	*RCN1*	88	127	78	293	201	62	41	218	522	815
Setaria italica CEN NW 004675967	*RCN1*	86	143	86	315	201	62	41	218	522	837
Setaria italica CEN NW 004675967.1	*RCN1*	134	243	137	514	201	62	41	218	522	1036
Setaria italica tfl1 NW 004675968	*RCN1*	86	151	79	316	201	62	41	218	522	838
Sorghum bicolor SORBIDRAFT 05g003200 NC 012874	*RCN1*	86	149	86	321	201	62	41	218	522	843
Sorghum bicolor SORBIDRAFT 08g003210 NC 012877	*RCN1*	82	149	81	312	201	62	41	218	522	834
Brachypodium distachyon LOC100844906 NC 016133	*RCN2*	151	301	190	642	201	62	41	218	522	1164
Oryza sativa Os02g0531600 NC 008395.2	*RCN2*	117	261	265	643	201	62	41	218	522	1165
Setaria italica CEN NW 004675961	*RCN2*	91	291	196	578	201	62	41	218	522	1100
Sorghum bicolor SORBIDRAFT 04g021650 NC 012873	*RCN2*	-	296	214	510	263	-	41	218	522	1032

Brachypodium distachyon LOC100835593 NC 016135	*RCN3*	108	292	130	530	201	62	41	218	522	1052
Oryza sativa Os04g0411400 NC 008397	*RCN3*	179	613	215	1007	201	62	41	218	522	1529
Sorghum bicolor SORBIDRAFT 06g015490 NC 012875	*RCN3*	170	187	152	509	201	62	41	218	522	1031
Amborella trichopoda TFL1 GSVIVT01033829001	Basal	651	655	138	1444	201	62	41	212	516	1960

Table S3 Types and numbers of putative *cis*-acting elements in intron 1~3 of angiosperm *TFL1/CEN/RCNs* paralogs

Taxa	Paralog	TATA box			ABRE			ARE			Box I			CAAT box			G box			MBS			ACG box			CCAAT box			CArG box			LFY bs			WRKY			Total
		I1	I2	I3	I1	I2	I3	I1	I2	I3	I1	I2	I3	I1	I2	I3	I1	I2	I3	I1	I2	I3	I1	I2	I3	I1	I2	I3	I1	I2	I3	I1	I2	I3	I1	I2	I3	
Lindera megaphylla TFL1 2042a2	Magnoliids	0	0	0	0	0	0	3	0	0	0	3	0	0	7	6	0	0	0	0	1	0	0	0	1	0	7	0	1	5	1	0	0	0	0	6	9	50
Liriodendron TFL1	Magnoliids	0	0	1	0	0	0	0	0	0	0	0	0	1	15	6	0	0	1	0	0	0	0	1	1	0	2	1	0	2	1	0	0	2	0	26	8	68
Arabidopsis lyrata tfl1 NW 003302550	*TFL1*	1	1	0	0	0	0	0	0	0	0	0	0	2	3	0	0	0	0	0	0	0	0	0	0	0	0	0	0	0	0	0	0	0	2	1	0	10
Arabidopsis thaliana TFL1 NC 003076.8	*TFL1*	1	0	0	0	0	0	1	0	0	0	0	0	1	1	0	0	0	0	0	0	0	0	0	0	0	0	0	0	0	0	0	0	0	0	1	0	5
Citrus clementina TFL1 NW 006262139	*TFL1*	0	1	0	0	0	0	0	0	0	0	0	0	1	3	1	0	0	0	0	0	0	0	0	0	0	1	0	0	0	0	0	0	0	0	4	1	12
Fragaria vesca tfl1 NC 020496	*TFL1*	0	0	0	0	0	0	0	0	0	0	0	0	1	5	0	0	1	0	0	0	0	0	0	0	0	1	0	0	0	0	0	0	0	0	5	0	13
Medicago truncatula TFL1 NC 016413.1	*TFL1*	0	0	0	0	0	0	0	0	0	0	0	0	1	0	0	0	0	0	0	0	0	0	0	0	0	0	0	0	0	0	0	0	0	1	0	1	3
Arabidopsis lyrata ATC NW 003302551	*CEN*	0	0	0	1	0	0	0	0	0	1	0	0	0	2	0	1	0	0	1	0	0	0	0	0	0	0	0	1	0	0	0	0	0	3	1	0	11
Arabidopsis thaliana ATC NC 003071.7	*CEN*	1	0	0	1	0	0	0	0	0	1	0	0	0	1	0	1	0	0	1	0	0	0	0	0	0	0	0	0	0	0	0	0	0	4	1	0	11
Arabidopsis lyrata ATC NW 003302552	*CEN*	1	1	0	1	0	0	0	0	0	1	0	0	0	2	0	1	0	0	1	0	0	0	0	0	0	0	0	0	0	0	0	0	0	3	1	0	12
Citrus clementina CEN NW 006262022	*CEN*	0	0	0	0	0	0	0	0	0	0	0	0	0	2	1	0	1	0	0	0	0	0	0	0	0	0	0	0	1	1	0	0	0	0	2	0	8
Fragaria vesca tfl1 NC 020492	*CEN*	0	0	0	0	0	0	0	0	0	0	0	0	1	0	0	0	0	0	0	1	0	0	0	0	1	0	0	0	0	0	0	0	0	0	0	1	4
Medicago truncatula TFL1 NC 016408.1	*CEN*	0	1	0	0	0	0	0	0	0	0	0	0	1	9	1	0	0	0	0	0	0	0	0	0	0	3	0	0	1	2	0	0	0	1	7	1	27
Setaria italica CEN NW 004675967	*RCN1*	0	0	0	0	0	0	0	0	0	0	0	0	0	0	0	0	0	0	0	0	0	0	0	0	0	0	0	0	0	0	0	0	0	1	0	0	1
Sorghum bicolor SORBIDRAFT 05g003200 NC 012874	*RCN1*	0	0	0	0	0	0	0	0	0	0	0	0	0	1	2	0	0	0	0	0	0	0	0	0	0	0	0	0	0	0	0	0	0	2	1	0	6
Setaria italica tfl1 NW 004675968	*RCN1*	0	0	0	0	0	0	0	0	0	0	1	0	1	0	0	0	0	0	0	0	0	0	0	0	1	0	0	0	0	0	0	0	0	1	2	1	7
Sorghum bicolor SORBIDRAFT 08g003210 NC 012877	*RCN1*	0	0	0	0	0	0	0	0	0	0	0	0	0	1	1	0	0	0	0	0	0	0	0	0	0	0	0	0	0	0	0	0	0	2	1	0	5
Brachypodium distachyon LOC100844217 NC 016134	*RCN1*	0	0	0	0	0	0	0	0	0	0	0	0	1	1	0	0	0	0	0	0	0	0	0	0	0	1	0	0	0	0	0	0	0	0	1	0	4
Oryza sativa Os12g0151900 NC 008404.2	*RCN1*	0	0	0	0	0	0	1	0	0	0	0	0	0	1	0	0	0	0	0	0	0	0	0	0	0	1	0	0	0	0	0	0	0	0	1	1	5
Setaria italica CEN NW 004675967.1	*RCN2*	0	1	0	0	0	0	0	0	0	0	0	0	0	1	1	0	0	0	0	0	0	0	0	0	0	0	0	0	0	1	0	0	0	0	3	0	7
Sorghum bicolor SORBIDRAFT 06g015490 NC 012875	*RCN2*	0	1	0	0	0	0	0	0	0	0	0	0	0	0	2	0	0	0	0	0	0	0	0	0	0	0	0	1	0	0	0	0	0	0	3	0	7
Oryza sativa Os04g0411400 NC 008397	*RCN2*	0	1	0	0	0	0	0	0	0	0	0	0	0	6	1	0	0	0	0	0	0	0	0	0	0	0	0	0	2	0	0	0	0	2	4	0	16
Brachypodium distachyon LOC100835593 NC 016135	*RCN2*	0	0	0	0	0	0	0	0	0	0	0	0	0	2	0	0	0	0	0	0	1	0	0	0	0	1	0	0	1	0	0	0	0	1	3	1	10
Brachypodium distachyon LOC100844906 NC 016133	*RCN3*	0	0	0	0	0	0	0	0	0	0	0	0	1	4	0	0	0	0	0	0	0	0	0	0	0	1	0	0	0	0	0	0	0	0	2	0	8
Oryza sativa Os02g0531600 NC 008395.2	*RCN3*	0	0	0	0	0	0	0	0	0	0	0	0	0	2	5	0	0	0	0	0	0	0	0	0	0	0	0	0	0	0	0	0	0	0	5	2	14
Setaria italica CEN NW 004675961	*RCN3*	0	0	0	0	0	0	0	0	0	0	0	0	0	1	0	0	0	0	1	0	0	0	0	0	0	0	0	0	1	1	0	0	0	0	3	0	7
Sorghum bicolor SORBIDRAFT 04g021650 NC 012873	*RCN3*	0	0	0	0	0	0	0	0	0	0	0	0	2	1	0	0	0	0	0	0	0	0	0	0	0	0	0	1	0	0	0	0	0	1	1	0	6
Amborella trichopoda TFL1 GSVIVT01033829001	basal	0	0	0	0	0	0	0	0	0	0	1	0	2	4	2	0	0	0	0	0	0	0	0	0	0	1	0	0	1	0	0	0	0	4	4	0	19
Total		4	7	1	3	0	0	5	0	0	3	5	0	16	75	29	3	2	1	4	2	1	0	1	2	2	19	1	4	14	7	0	0	2	28	89	26	

I1, I2, I3 indicate the intron 1, intron 2, and intron 3, respectively.

[a] LFY binding site

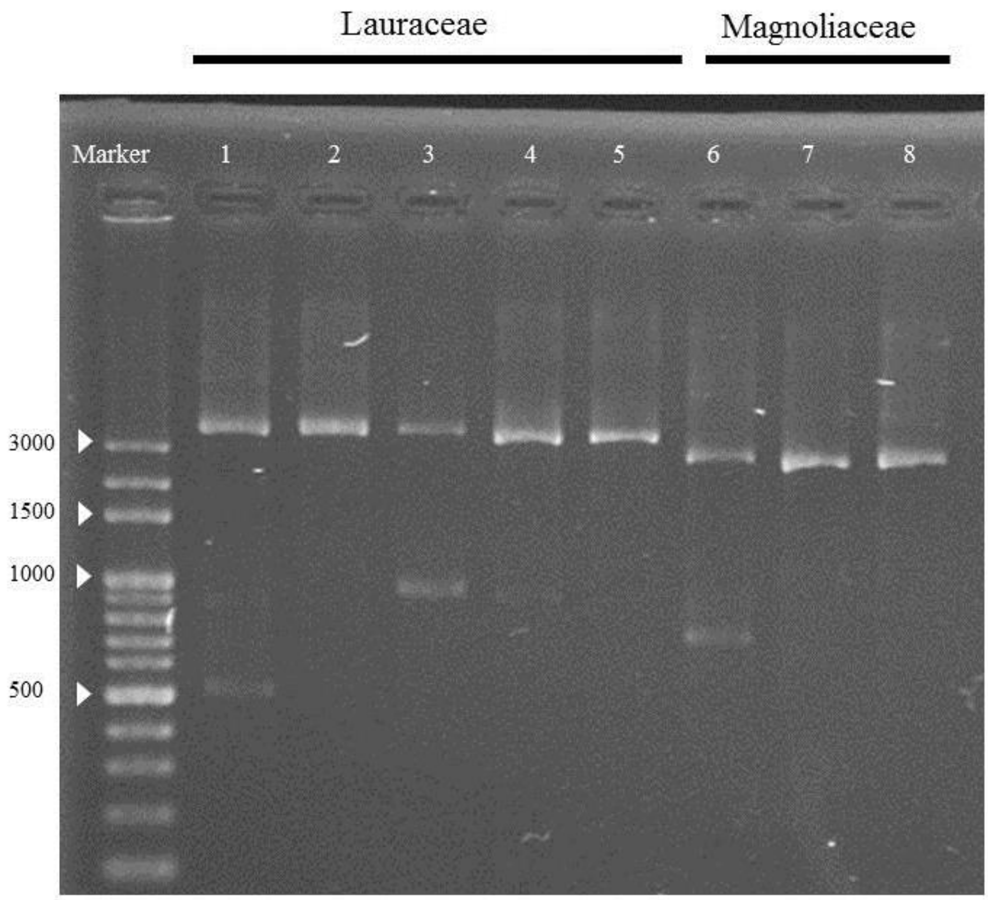

Figure S1 PCR products amplified from Lauraceae and Magnoliaceae member using primers of *TFL1*-like. Marker: 100-bp ladder. 1: *Machilus japonica*; 2: *Machilus obovatifolia*; 3: *Cinnamomum kanehirai*; 4: *Cinnamomum macrostemon*; 5: *Persea americana*; 6: *Parakmeria yunnanensis*; 7: *Michelia figo*; 8. *Magnolia denudate*. Arrows indicated different size (bp).

Bands with size lower than 1000-bp are non-specific product.

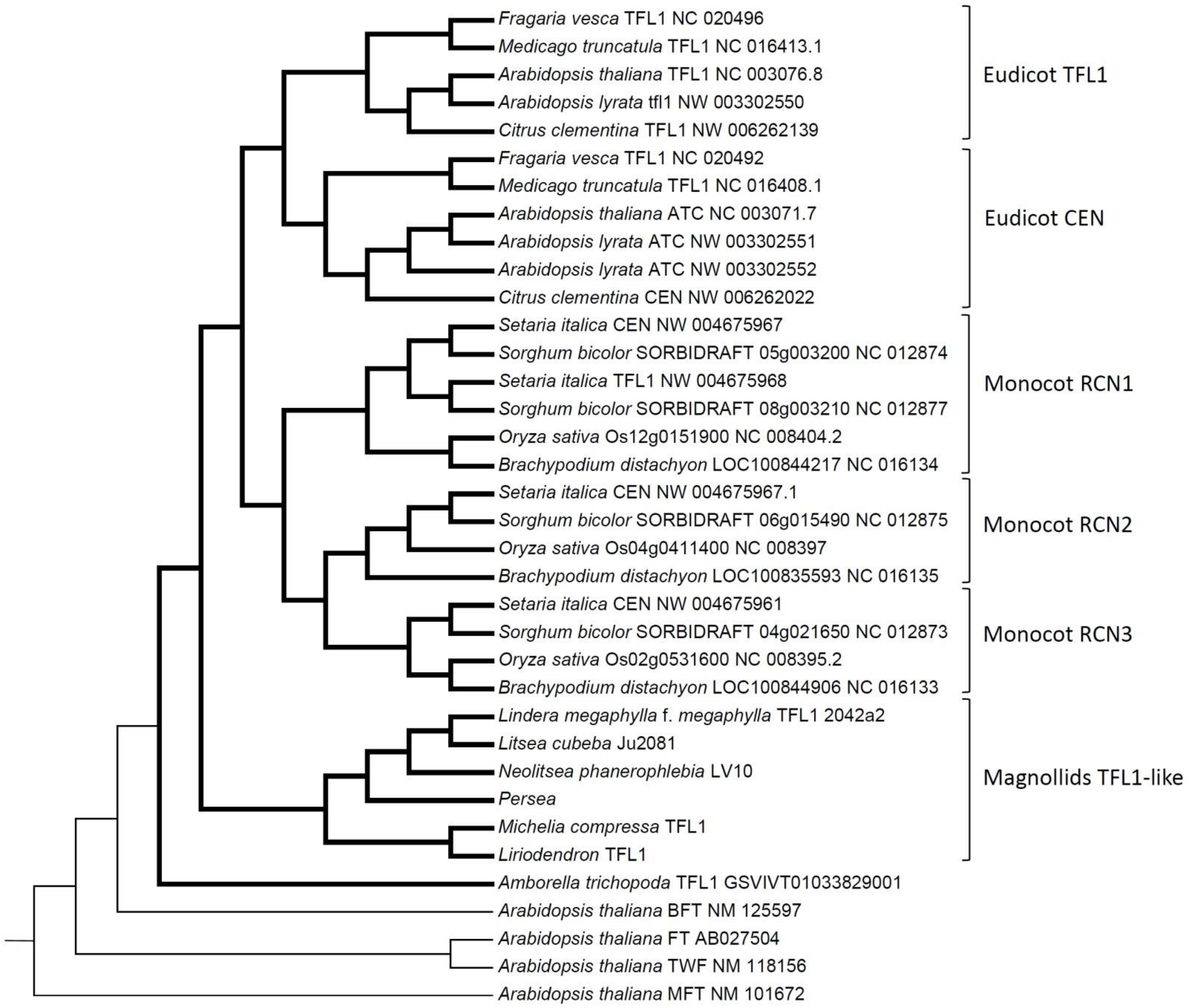

Figure S2 Hypothetical tree topology of angiosperm *TFL1/CEN/RCNs* paralogs. The full tree was used for the DIVERGE analysis and the bold portion was used as the prior for PAML analysis.

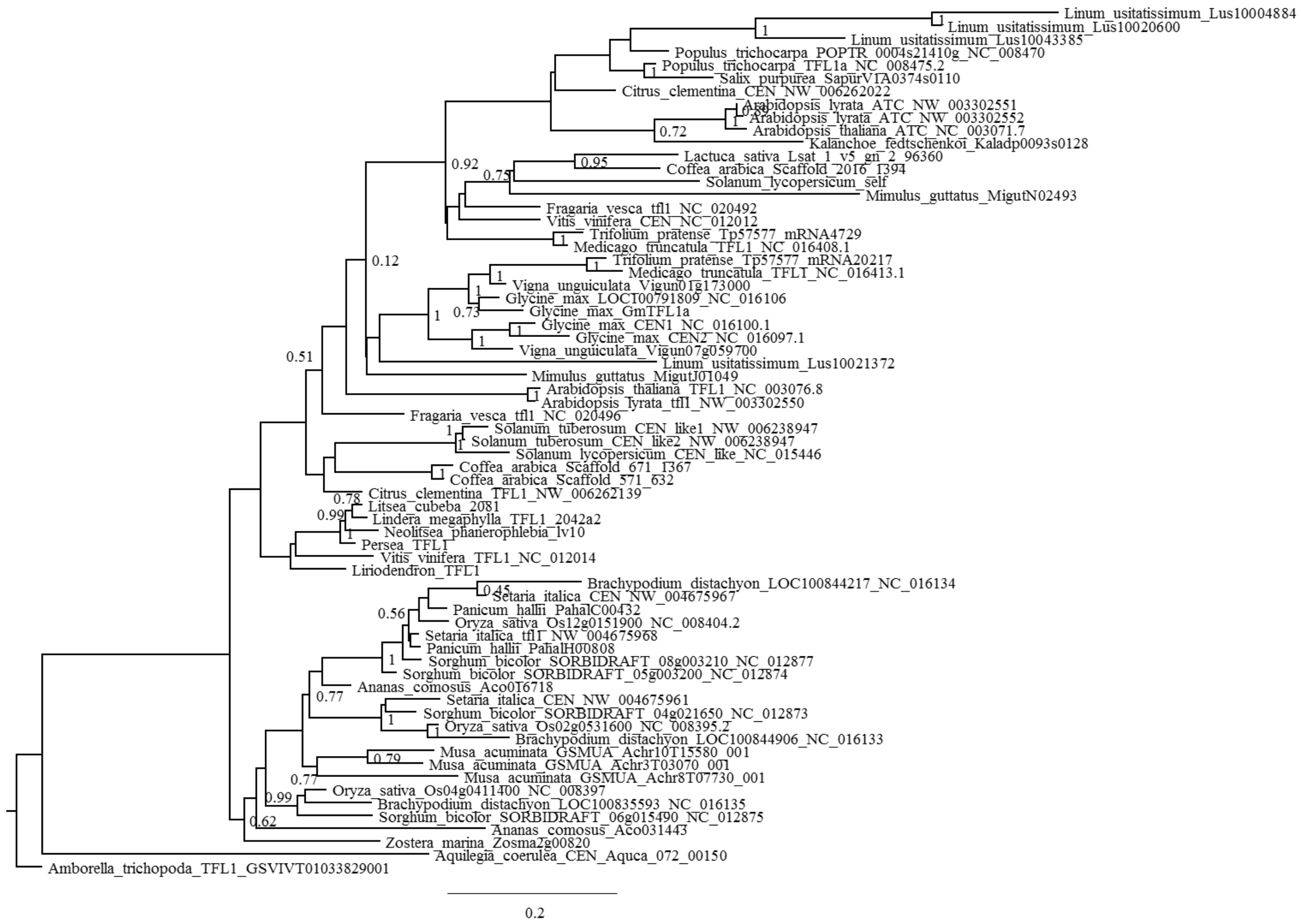

Figure S3 Gene tree of angiosperm *TFL1/CEN/RCNs* paralogs reconstructed by Bayesian inference. Values of the nodes are posterior probabilities for branch supports.
